# Long-Term Differential Changes in Mouse Intestinal Metabolomics after γ and Heavy Ion Radiation Exposure

**DOI:** 10.1371/journal.pone.0087079

**Published:** 2014-01-27

**Authors:** Amrita K. Cheema, Shubhankar Suman, Prabhjit Kaur, Rajbir Singh, Albert J. Fornace, Kamal Datta

**Affiliations:** 1 Department of Biochemistry and Molecular & Cellular Biology and Lombardi Comprehensive Cancer Center, Georgetown University, Washington, D.C., United States of America; 2 Center of Excellence In Genomic Medicine Research (CEGMR), King Abdulaziz University, Jeddah, Saudi Arabia; Enea, Italy

## Abstract

Tissue consequences of radiation exposure are dependent on radiation quality and high linear energy transfer (high-LET) radiation, such as heavy ions in space is known to deposit higher energy in tissues and cause greater damage than low-LET γ radiation. While radiation exposure has been linked to intestinal pathologies, there are very few studies on long-term effects of radiation, fewer involved a therapeutically relevant γ radiation dose, and none explored persistent tissue metabolomic alterations after heavy ion space radiation exposure. Using a metabolomics approach, we report long-term metabolomic markers of radiation injury and perturbation of signaling pathways linked to metabolic alterations in mice after heavy ion or γ radiation exposure. Intestinal tissues (C57BL/6J, female, 6 to 8 wks) were analyzed using ultra performance liquid chromatography coupled with electrospray quadrupole time-of-flight mass spectrometry (UPLC-QToF-MS) two months after 2 Gy γ radiation and results were compared to an equitoxic ^56^Fe (1.6 Gy) radiation dose. The biological relevance of the metabolites was determined using Ingenuity Pathway Analysis, immunoblots, and immunohistochemistry. Metabolic profile analysis showed radiation-type-dependent spatial separation of the groups. Decreased adenine and guanosine and increased inosine and uridine suggested perturbed nucleotide metabolism. While both the radiation types affected amino acid metabolism, the ^56^Fe radiation preferentially altered dipeptide metabolism. Furthermore, ^56^Fe radiation caused upregulation of ‘prostanoid biosynthesis’ and ‘eicosanoid signaling’, which are interlinked events related to cellular inflammation and have implications for nutrient absorption and inflammatory bowel disease during space missions and after radiotherapy. In conclusion, our data showed for the first time that metabolomics can not only be used to distinguish between heavy ion and γ radiation exposures, but also as a radiation-risk assessment tool for intestinal pathologies through identification of biomarkers persisting long after exposure.

## Introduction

Space travel beyond low earth orbit exposes astronauts to radiation from solar particle events (SPE), galactic cosmic radiation (GCR), and earth’s magnetosphere (Van Allen belt) [1]. While high-energy protons constitute a major part of sporadically occurring SPE, heavy ions such as ^56^Fe, ^28^Si, ^16^O, and ^12^C are the major contributors to the dose equivalent in GCR, which is ubiquitous in space [2]. Heavy ion radiation with its high linear energy transfer (high-LET) characteristics is known not only to cause dense ionization events along its primary tract but also to generate greater numbers of secondary ionization tracts (delta rays) in the traversed tissues relative to low-LET γ radiation [3]–[6]. During prolonged space missions such, as a mission to Mars, astronauts could receive a cumulative radiation dose that has the potential for long-term deleterious effects on human health [1], [7]. However, there is a paucity of *in vivo* long-term follow up data at the molecular level to help understand persistent metabolic consequences of radiation including space radiation on critical tissues such as intestine. Indeed, current uncertainties in the assessment of risk to gastrointestinal (GI) tissues restrain the permissible duration of astronauts’ stay in space. Both small and large intestine are involved in the absorption of essential nutrients and radiation exposure has been reported to perturb intestinal cell physiology to affect nutrient absorption and human health [8]–[10]. Furthermore, we have earlier shown persistent oxidative stress in the intestinal epithelial cells even twelve months after heavy ion ^56^Fe radiation exposure [11]. Consequently, heavy ion radiation due to its higher relative biological effectiveness (RBE) compared to γ and proton radiation [12], [13] is expected to have greater potential to adversely affect intestinal cell metabolic activities and thus raising human health concern during prolong space travel.

Radiation exposure has been associated with a myriad of cellular responses at the genomic, proteomic, as well as at the metabolic level [14], [15]. Differential metabolic responses between radiation exposed and non-exposed cells has allowed identification of metabolites, which has the potential to serve as exposure-markers in the short term and as risk-markers in the long-term for chronic diseases such as cancer. Indeed, metabolomics is increasingly used towards understanding radiation exposure-associated pathophysiological changes and resultant disease processes in human [16]–[20]. Also, metabolomics adds an additional dimension to the ‘omics’ based systems biology approach not only to estimate disease risk but also to assess disease promotion and progression through biomarker identification for the target tissue. Metabolomics aims to separate, identify, and quantify small metabolites (<1800 Da) from cells, tissues, or bio-fluids such as serum and urine by combining the enhanced analytical technology and improved computational capacity of bioinformatics tools [18]. Unlike transcriptomics and proteomics, metabolomics allows insight into the ongoing biological processes after radiation exposure and is considered an end-response to changes at the gene and protein expression patterns. Radiation metabolomics is a rapidly growing area of research targeted mostly towards development of minimally invasive radiation biodosimetry using biofluids such as serum and urine and tissues, with an aim to discern radiation exposure and dose in a given population during a radiological event [14], [21]–[23]. Acute radiation exposure has been shown to modulate intestinal cell metabolism affecting cell viability and metabolomics studies have identified a number of metabolites including tryptophan, glutamic acid, and taurocholic acid involved in acute GI injury [15], [20]. However, radiation-induced long-term intestinal metabolic changes reflecting pathophysiological changes associated with chronic human diseases have not been clearly defined.

Radiation is known to promote oxidative stress and inflammation through alterations in biochemical pathways leading to acute effects such as cell death and GI mucositis and chronic effects such as persistent inflammation, cellular transformation, and cancer [15], [24]. Inflammation has been implicated not only in chronic intestinal ailments such as Crohn’s disease and ulcerative colitis compromising nutrient absorption but also in colorectal cancer [25]–[27]. A major player in intestinal inflammation is prostaglandin E2 (PGE2), which unlike cytokines is an arachidonic acid metabolite [28]. The membrane-bound cyclooxygenases (COX1 and 2) convert arachidonic acid to PGH2, which is then converted to PGE2 by PGE synthase 1 (PGES1), and 15-hydroxy PG dehydrogenase further metabolizes PGE2 to 13,14-dihydro-15-keto-PGE2 for excretion [28], [29]. While COX1 is constitutively expressed to produce prostaglandins required for a number of physiological functions through its action on smooth muscle, bone, ovary, brain, and GI tract, COX2 and PGES1 are induced by inflammatory cytokines such as interleukin 1β (IL1β) and higher expression of COX2 and PGES1 has been reported in inflammatory bowl disease as well as in CRC [28]. Apart from PGE2, PGH2 also acts as a precursor molecule for PGD2 and thromboxane A2 (TXA2) both of which have been reported to play roles in inflammatory processes via their respective G-protein coupled receptors [28], [30], [31]. On the contrary PGE2 acting via four different G-protein coupled E prostanoid (EP) receptors activate a number of kinases such as extracellular signal regulated kinase (ERK), c-Jun NH2-terminal kinase (JNK), and PI3K/Akt to promote cell proliferation [28]. Low-LET γ radiation exposure has been shown to upregulate COX2 and consequently PGE2, which have been implicated in cell survival and tumor recurrence [32,32]. Considering lack of *in vivo* molecular pathway data and risk-prediction model for GI tissues after heavy ion radiation exposure, the current study undertakes a systems biology approach through metabolomics and proteomics to assess long-term changes in the murine GI tract two months after exposure to 1.6 Gy of heavy ion ^56^Fe radiation and results were compared to a dose of (2 Gy) γ radiation commonly used in fractionated radiotherapy. We show that the metabolic profile of each study group was distinct and principal component analysis (PCA) showed spatial separation of the control, γ, and ^56^Fe irradiated groups. While γ radiation exposure led to upregulation of ‘bile acid biosynthesis’ and ‘lanosterol biosynthesis’, ^56^Fe radiation exposure caused increased ‘prostanoid biosynthesis’ and ‘ecosanoid signaling’, which are implicated in inflammation. Immunoblot and immunohistochemistry analysis showed greater effects on proliferative pathways after ^56^Fe radiation relative to γ radiation.

## Materials and Methods

### Ethics statement

Animal facilities at Brookhaven National Laboratory (BNL) and Georgetown University (GU) are AAALACI (Association for Assessment and Accreditation of Laboratory and Animal Care International) accredited facilities. All animal procedures were performed as per protocol approved by the BNL and GU animal care and use committee. The full name of the animal protocol approval committee at Georgetown is Georgetown University Animal Care and Use Committee (GUACUC) and the approved protocol number is #13-021. The full name of the animal protocol approval committee at BNL is BNL Animal Care and Use Committee (BNLACUC) and the approved protocol number is #345. Mice were housed in autoclaved cages and bedding materials in a separate room with 12-h dark and light cycle maintained at 22 °C in 50% humidity. All animals were provided certified rodent diet with filtered water ad libitum and CO_2_ asphyxiation was used for euthanasia. Any mouse with declining health determined by using the parameters such as hunched posture, ruffled fur, diarrhea, reduced activity, and weight loss (>15%) was euthanized by CO_2_ asphyxiation and was excluded from the specific study group. Our research followed Guide for the Care and Use of Laboratory Animals, prepared by the Institute of Laboratory Animal Resources, National Research Council, and U.S. National Academy of Sciences.

### Chemicals and reagents

Liquid chromatography/mass spectrometry (LC/MS)-grade acetonitrile (ACN), water, and methanol were purchased from Fisher Scientific (Pittsburg, PA, USA). High purity formic acid (99%) was purchased from Thermo Scientific (Rockford, IL, USA). Phenylalanine, glutathione, guanosine, adenine, asparatate, 5-hyroxytryptophan, leucyl-leucine, uridine, dethiobiotin, oxidized glutathione, glycyl-leucine, S-ATPA, PGE2, 17-hydroxyprogesterone, creatinine, pyruvate, debrisoquine, 4-nitrobenzoic acid (4-NBA) were purchased from Sigma Aldrich (St. Louis, MO, USA). All the reagents and chemicals used were LC/MS grade.

### Mice and radiation

Female C57BL/6J mice (6 to 8 wks) were purchased from Jackson Laboratories (Bar Harbor, ME, USA) and were directly shipped to BNL animal facility one week prior to radiation exposure. Mice (n = 8 mice per group) were randomly assigned to study groups and ^56^Fe-irradiated (1.6 Gy; energy-1000 MeV/nucleon; LET-148 keV/µm) at the NASA Space Radiation Laboratory (NSRL) in BNL and a ^137^Cs source was used for 2 Gy γ irradiation. The ^56^Fe radiation dose of 1.6 Gy is equitoxic to 2 Gy γ radiation and was calculated using a relative biological effectiveness (RBE) factor of 1.25 determined earlier [12]. Mice were irradiated in small transparent rectangular Lucite boxes (7.6 cm×3.8 cm×3.8 cm) with multiple holes for air circulation. The NSRL physics team determined heavy ion radiation dosimetry and mice were exposed to constant LET by placing them at the entrance plateau region of the Bregg curve [1], [33]–[35]. Mice were shipped from BNL to GU animal facility on the day after irradiation early in the morning in a temperature-controlled environment along with the respective sham irradiated control groups for same day delivery.

### Intestinal tissue harvesting and sample preparation

Approved protocol was used to euthanize mice 2-month after radiation exposure using CO_2_ asphyxiation procedure. Small intestine was surgically removed, flushed with phosphate buffered saline (PBS), tissue sections from the jejuno-ilial region were flash frozen in liquid N_2_, and stored at –80°C for further processing. For metabolomics analysis, intestinal tissue samples were processed as per protocol described earlier [20]. Briefly, uniform tissue sections were homogenized in buffer containing 50% methanol. The protein was precipitated by the addition of acetonitrile (100% volume/volume). The samples were centrifuged and the supernatant was transferred to a fresh vial and dried under vacuum and subsequently resuspended in buffer containing 5% methanol and 95% water for mass spectrometry (MS) analysis. The pellets were resuspended in RIPA buffer, incubated on ice for 15 min, and centrifuged. Protein estimation was performed using Bradford method [36]. Total protein concentration was used to normalize the raw data.

### Ultra performance liquid chromatography and electrospray quadrupole time-of-flight mass spectrometry (UPLC-QTOF-MS)

Metabolomic profiling was performed using UPLC-QTOF-MS as described previously [20]. Briefly, 5 μl of each sample was injected onto a reverse-phase 2.1×50 mm Acquity 1.7 µm C18 column (Waters Corporation, Milford, MA, USA) using an Acquity UPLC system with a gradient mobile phase consisting of 2% acetonitrile in water containing 0.1% formic acid (solution A) and 2% water in acetonitrile containing 0.1% formic acid (solution B). Each sample was resolved for 10 min at a flow rate of 0.5 ml/min. The gradient consisted of 100% A for 0.5 min then a ramp of curve 6 to 60% B from 0.5 min to 4.0 min, then a ramp of curve 6 to 100% B from 4.0–8.0 min, hold at 100% B until 9.0 min, then a ramp of curve 6 to 100% A from 9.0 min to 9.2 min, followed by a hold at 100% A until 10 min. The column eluent was introduced directly into the mass spectrometer by electrospray. Mass spectrometry was performed on a Q-TOF instrument (QTOF Premiere, Waters, Columbia, MD, USA), operating in either negative (ESI-) or positive (ESI+) electrospray ionization mode with a capillary voltage of 3200 V and a sampling cone voltage of 20 V in negative mode and 35 V in positive mode. The desolvation gas flow was set to 800 liters/h and the temperature was set to 350 °C. The cone gas flow was 25 liters/h, and the source temperature was 120 °C. Accurate mass was maintained by introduction of lock spray interface of sulfa-dimethoxine (*m/z*  =  311.0814 [M+H]^+^ or 309.0658 [M-H]^−^) at a concentration of 250 pg/µl in 50% aqueous acetonitrile and a rate of 150 µl/min. Data were acquired in centroid mode from 50 to 850 *m/z* in MS scanning.

### Metabolomics data analysis

UPLC-QTOF-MS data were pre-processed using XCMS software. The data were normalized to the ion intensity of the internal standards and protein concentration. The normalized data sets were analyzed by unsupervised Principal Component Analysis (PCA) as well as supervised Orthogonal Projections to Latent Structures Discriminant Analysis (OPLS-DA) using the SIMCA-P v11.5 (Umetrics Inc., Umea, Sweden). Candidate features with high correlation values and positioned furthest from the point of origin in the upper right and lower left quadrants of the S-plot were chosen for further characterization. Quantitative descriptors of model quality for the OPLS-DA models included R^2^ (explained variation of the binary outcome: sham vs irradiated) and Q^2^ (cross-validation based predicted variation of the binary outcome). We used score plots to visualize the group discriminating properties of the OPLS-DA models, and also S-plots for putative biomarker identification by visualization of the OPLS-DA loadings on the predictive score. Selection of features based on the OPLS-DA model used a p (correlation) cut-off of 0.8, as reported previously [37], [38]. The features selected via OPLS-DA were subjected to accurate mass based search using human metabolome database (HMDB), Madison Metabolomics Consortium Database (MMCD), and Lipid Maps databases.

### Mass-based putative metabolite identification and Ingenuity Pathway Analysis

Metabolite mass data obtained from UPLC-TOF-MS analysis were uploaded to Metabosearch [39], which searched the metabolomic databases for mass-based putative identification of compounds. Significantly perturbed putative metabolites with Kegg ID were uploaded to Ingenuity Pathway Analysis (IPA, Ingenuity Systems Inc., Redwood City, CA, USA) for understanding how the dataset relates to biological functions and also for recognizing which signaling pathways are associated with the experimental dataset. IPA correlates the Ingenuity Pathways Knowledge Base (IPKB) and the uploaded experimental dataset to recognize biological functions that are significantly associated with the metabolomics data. IPA determines level of significance displayed as scores by right-tailed Fisher's exact test and a score of 2 indicates that there is a 1 in 10^2^ chance that the focus molecules are together in a network due to random chance alone. IPA also generates canonical signaling pathways significantly (p<0.05 presented as corresponding –log(p-value) of 1.3) associated with the metabolomics dataset. The putative identifications of biologically relevant selected metabolites were confirmed using tandem mass spectrometry wherein the fragmentation pattern and retention times of the parent ions in the tissue extract were matched with the standard compound.

### Immunoblot analysis

Frozen intestinal tissue samples from 5 mice were pooled, homogenized in ice-cold lysis buffer (0.5% sodium deoxycholate; 0.5% NP-40; 10 mM EDTA in PBS) containing protease inhibitor cocktail (Sigma), and centrifuged at 12000xg at 4 °C for 15 min. Protein was estimated in the supernatant, and separated by sodium dodecyl sulphate-polyacrylamide gel electrophoresis (SDS-PAGE), and transferred onto polyvinylidene fluoride (PVDF) membrane. The membrane was blocked with 5% non-fat milk (Bio-Rad, Hercules, CA, USA) in tris-buffered saline with 0.1% Tween (TBST), and incubated with appropriate primary antibodies (anti-PGE2 receptor, Cat#MAL12677, Thermo Fisher Scientific, dilution-1∶500; anti-PI3K/p85, Cat#MAI74183, Thermo Fisher Scientific, dilution-1∶500; anti-COX2, Cat#sc1745, Santa Cruz Biotechnology, Dallas, TX, dilution-1∶500; anti-β-actin, Cat#sc47778, Santa Cruz Biotechnology, dilution-1∶2500; anti-phosho-JNK, Cat#9251S, Cell Signaling Technology, Danvers, MA, dilution-1∶1000; anti-PGE2 synthase, Cat#160140, Cayman Chemicals, Ann Arbor, MI, dilution-1∶250). After appropriate washing steps, the membranes were incubated with horseradish peroxidase (HRP) conjugated secondary antibody and enhanced chemiluminescence (ECL) detection system (Cat# 34080, Thermo Fisher Scientific) was used for developing the immunoblots. Photographic films were used for images capture and scanned immunoblot images were used for densitometric quantification by ImageJ v1.46 software and representative images are shown in the results. Band intensity was normalized to β-actin band intensity in respective columns and results are expressed as mean ± standard error of mean (SEM).

### Immunohistochemistry for Ki67

Sections of intestine from the jejunal-ilial area were flushed with phosphate buffered saline (PBS), fixed in 10% buffered formalin, paraffin embedded, and 4 μm sections were made. Control and experimental sections were deparaffinized, antigen retrieved in citrate buffer (pH 6.0; 20 min boiling), and stained with anti-Ki67 antibody (Cat#sc15402; dilution 1:50; Santa Cruz Biotechnology). Signal was detected using SuperPicture TM 3rd Gen IHC detection kit (Cat#87-9673; Invitrogen, Carlsbad, CA, USA). Stained sections were visualized under bright field microscopy and twenty random fields of vision (FOV) in each experimental and control group were captured for quantification. Images were quantified using color deconvolution and Image-based Tool for Counting Nuclei (ITCN) plug-ins of ImageJ v1.46 software as per protocol described earlier [11],[40], [41] and six mice in each group were used for quantification. Average number of Ki67 positive nuclei per animal is presented graphically and a representative image from each study group is presented in the results. Student’s t-test was used to determine level of significance (p<0.05) between two groups, and data is presented as mean ± standard error of mean (SEM).

## Results

### Differential alterations in intestinal tissue metabolites were observed after ^56^Fe radiation

**Figure 1:**
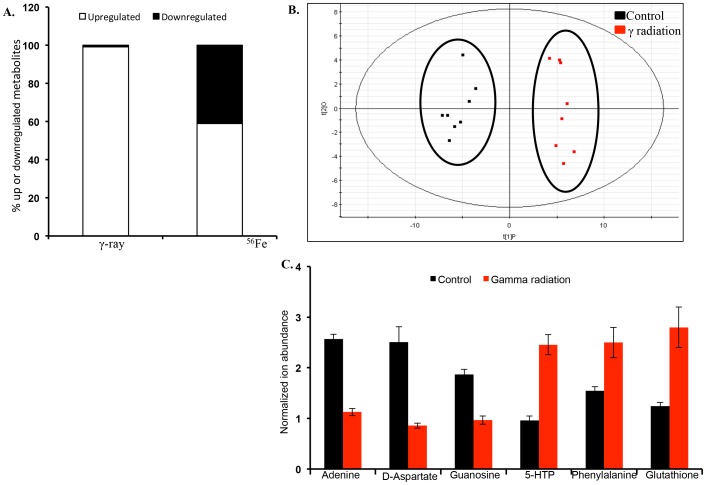
Intestinal tissue metabolites were differentially altered after γ and ^56^Fe radiation. A) Percent of metabolites up or downregulated after γ and ^56^Fe radiation. The features were extracted using XCMS and further selected based on the p-value cut off (p≤0.05) and fold change (≤ 0.5 or ≥1.5). B) Multivariate analysis shows distinct metabolic changes in γ irradiated mice in negative ionization mode. Scores plot depicting class separation between the sham and γ irradiated groups. C) Selective validated markers altered after γ irradiation are presented as normalized ion abundance relative to sham-irradiated control.

Metabolomic profiling of intestinal tissue in response to γ and ^56^Fe irradiation yielded a total of 6156 and 3082 features respectively. These features were further selected based on a significant fold change (≤ 0.5 or ≥1.5) and p-value (≤ 0.05) in the irradiated groups as compared to the sham-irradiated control groups. In the positive mode, a total of 1125 and 318 features were selected in γ and ^56^Fe irradiated groups respectively. In the negative mode, γ radiation exposure led to significant perturbation of 1222 features and ^56^Fe radiation resulted in perturbation of 346 features (**Table 1**). While in γ irradiated samples most of the features were upregulated (99.2%), in ^56^Fe irradiated groups 59% features were upregulated and 41% were downregulated (**Figure 1A**).

**Table 1:** Total number of features extracted using XCMS.

Mode	Total features*	Radiation	Significant features**
Positive	4214	γ-ray	1125
		^56^Fe	318
Negative	3668	γ-ray	1222
		^56^Fe	346

Features (metabolites) extracted using XCMS were further selected based on p-value (p≤0.05) and fold change (≤ 0.5 or ≥1.5) for further identification and validation. *Features extracted using XCMS. **Significance was determined based on p-value (p≤0.05) and fold change (≤ 0.5 or ≥1.5). Fold change - γ-ray/control and ^56^Fe/control.

### Metabolic profile led to distinct grouping of γ and ^56^Fe irradiated mice

**Figure 2:**
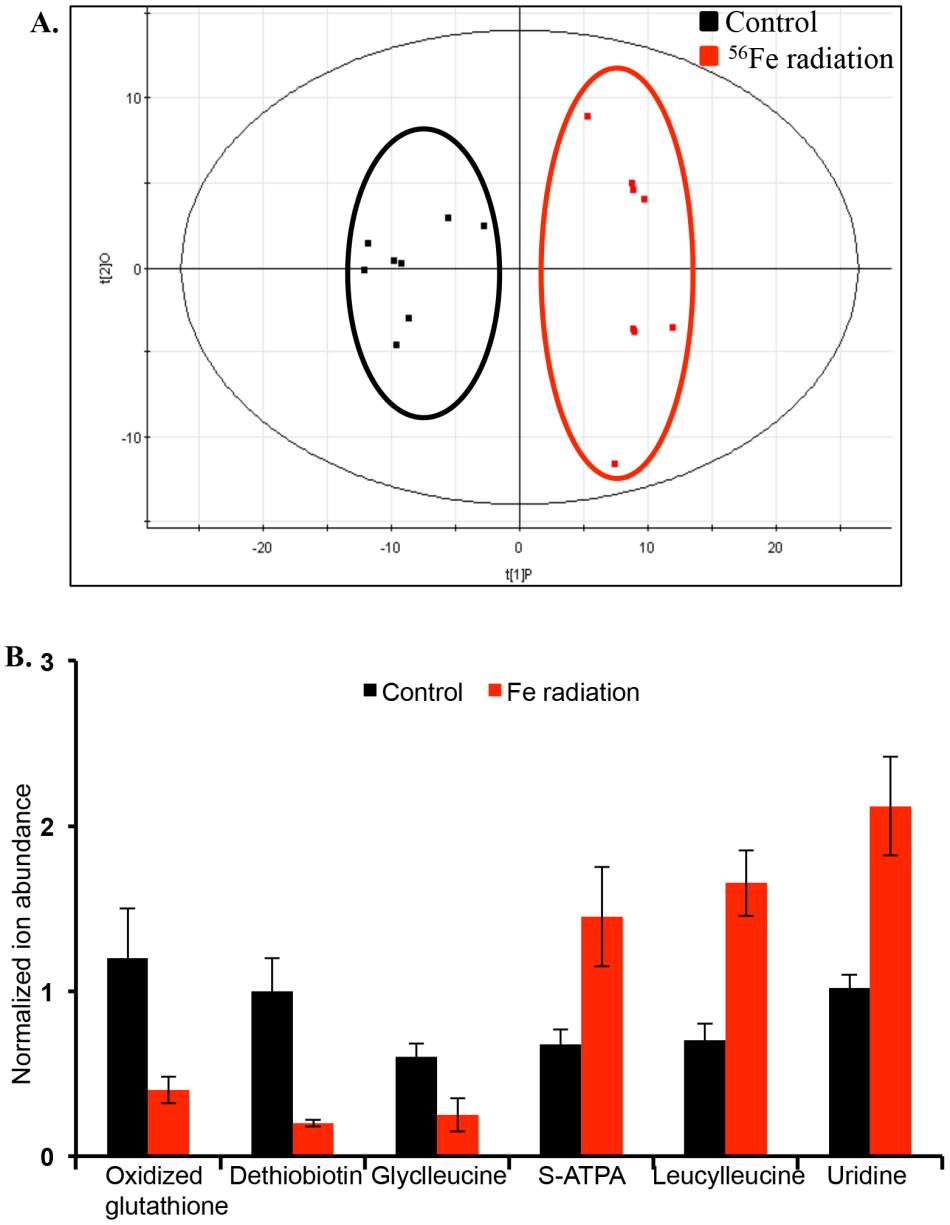
Multivariate analysis shows distinct metabolic changes in ^56^Fe-irradiated mice in negative ionization mode. A) Scores plot depicting class separation between the sham and ^56^Fe irradiated groups. B) Selective validated biomarkers altered after ^56^Fe irradiation are presented as normalized ion abundance relative to sham-irradiated control.

**Figure 3:**
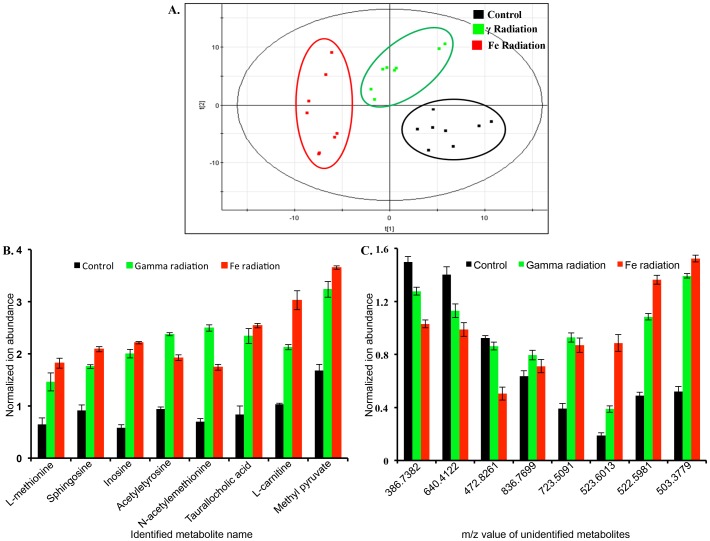
Multivariate analysis shows distinct metabolic profiles in sham, γ, and ^56^Fe irradiated mice in negative ionization mode. A) Scores plot showing class separation among the study groups. B) Trend plot for the selective putative biomarkers altered in both γ, and ^56^Fe irradiated groups relative to controls. C) Trend plot for the unidentified biomarkers showing statistically significant difference in the three groups are presented with m/z respective values.

**Figure 4:**
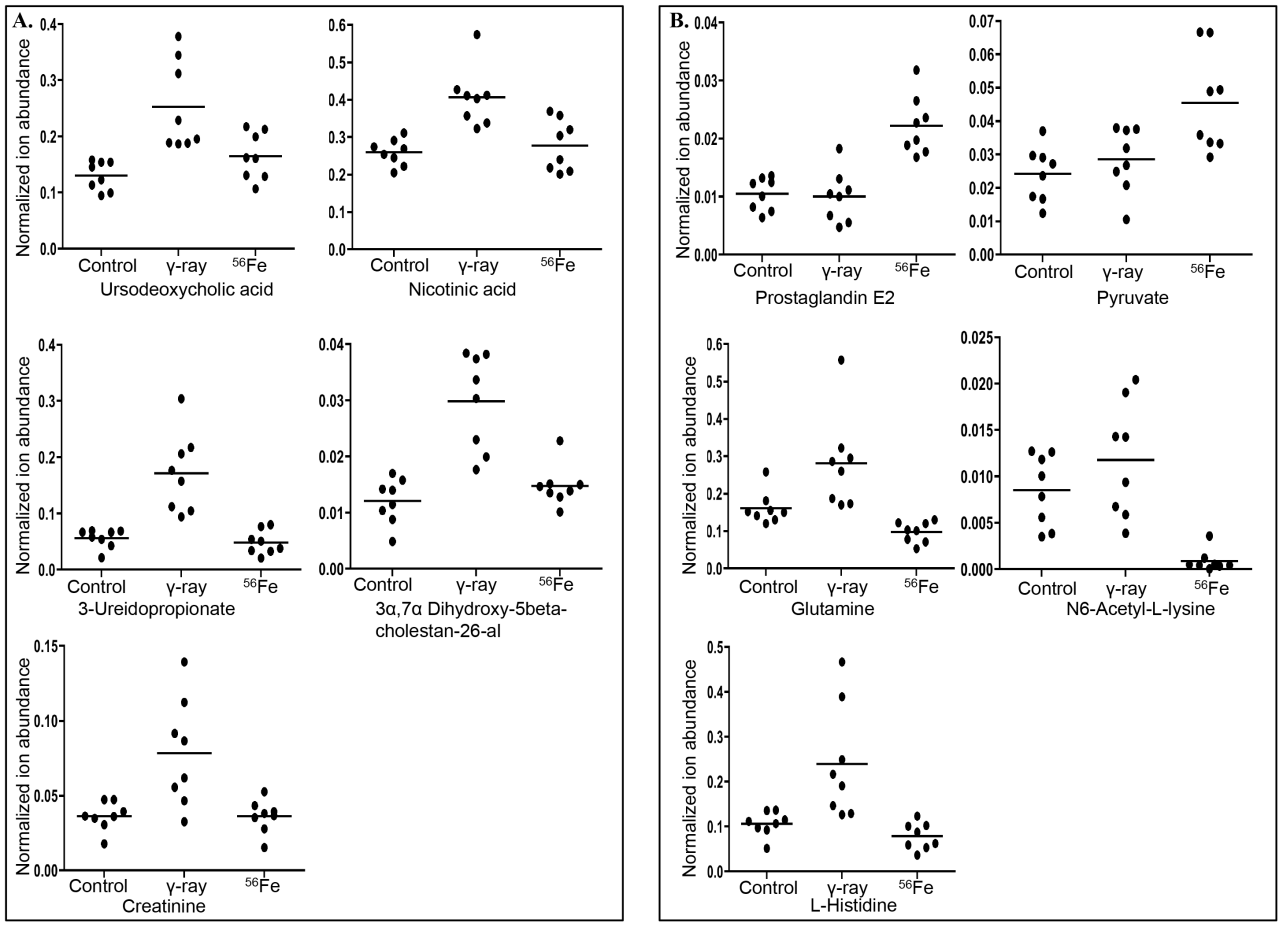
Selective biomarkers differentially altered after exposure to two types of radiation. A) Five biomarkers specifically altered after γ irradiation were selected based on their relevance to radiation damage and digestive system and presented as normalized ion abundance relative to control and ^56^Fe irradiated samples. B) Five biomarkers specifically altered after ^56^Fe irradiation were selected based on their relevance to radiation damage and digestive system and presented as normalized ion abundance relative to control and γ irradiated samples. Identity of creatinine, PGE2, and pyruvate were confirmed using tandem mass spectrometry and standard compounds.

Distinct separation between the sham- and γ-irradiated groups was observed in the negative mode and the R^2^ and Q^2^ for the OPLS-DA model were 0.94 and 0.77 respectively providing statistical support to the separation model (**Figure 1B**). Similar statistically significant group separation results were also observed in the positive mode (**Figure S1**). Selected metabolite identification was performed using tandem mass spectrometry wherein the fragmentation pattern and retention time for each compound from the tissue lysate was matched against the respective standard (**Table 2**). While phenyalanine, glutathione, and 5-hydroxy-tryptophan (5-HTP) levels were increased, the levels of guanosine, adenine, and aspartate were found to decrease in the intestinal tissue after γ radiation exposure (**Figure 1C**). The features obtained after ^56^Fe radiation were also subjected to OPLS-DA analysis, which showed distinct separation from the control group (**Figure 2A** **and Figure S2**). Selected metabolites from the ^56^Fe-irradiated group were confirmed by comparing fragmentation pattern and retention times against standard compounds (**Figure 2B** **and** **Table 3**). While tissue levels of oxidized glutathione, dethiobiotin, and glycyl-leucine were decreased, the levels of leucyl-leucine, S-ATPA, and uridine were significantly enriched after ^56^Fe irradiation, relative to controls (**Figure 2B**). Furthermore, PCA comparing control, γ, and ^56^Fe irradiated groups showed distinct profiles for each of the three groups emphasizing intrinsic differences in metabolic alterations (**Figure 3A**). A number of biologically relevant metabolites, which were altered in γ as well as ^56^Fe irradiated samples relative to control, were identified using database search for mass-based putative compounds (**Figure 3B**). While levels of these metabolites increased after exposure to both the radiation types, the changes were more pronounced after ^56^Fe radiation. Greater alteration in the levels of a number of unidentified metabolites was also observed after ^56^Fe radiation relative to γ radiation (**Figure 3C**). Additional biologically relevant but differentially regulated metabolites were also observed after γ and ^56^Fe radiation. While metabolites, ursodeoxycholic acid, nicotinic acid, 3-ureidopropionate, 3α,7α Dihydroxy-5beta-cholestan-26-al, and creatinine, were significantly increased after γ radiation, they remain unchanged after ^56^Fe radiation (**Figure 4A**). In contrast, PGE2, pyruvate, glutamine, N6-acetyl-L-lysine, and L-histidine were significantly altered after ^56^Fe radiation relative to control and γ radiation (**Figure 4B**). Identity of additional metabolites, creatinine, PGE2, 17-hydroxy progesterone, and pyruvate, were validated using tandem mass spectrometry and standard compounds (**Table 2** **and** **3**).

**Table 2:** Mass spectrometry confirmed identify of selected metabolites from γ irradiation.

Metabolites (Kegg ID)	Mode	m/z	RT	p-value	Major CID fragments
Phenylalanine (C02057)	POS	166.087	2.1	↑ (0.03)	149.0315, 120.0793,
					77.0384
Glutathione (C12392)	POS	308.092	0.3	↑ (0.02)	233.0577, 84.0439,
					76.0240
Guanosine (C00387)	POS	284.099	0.4	↓ (0.02)	110.0362, 135.0317,
					152.0589
Adenine (C00147)	NEG	134.049	0.3	↓ (0.03)	65.0159, 92.0268,
					107.0375
D-Aspartate (C16433)	NEG	132.036	0.3	↓ (0.04)	71.0152, 88.0405,
					115.0040
17-OH progesterone (C01176)	POS	331.244	6.7	↑ (0.05)	313.2296, 123.0854,
					109.0683
Glutathione (C00051)	POS	308.092	0.4	↑ (0.05)	233.0625, 162.0262,
					84.0478
Creatinine (C00791)	POS	114.067	0.3	↑ (0.05)	86.0700, 72.0526
5-Hydroxytryptophan (C01017)	NEG	219.077	0.6	↑ (0.03)	144.0480, 158.0646

Identity of biologically relevant selected metabolites from positive and negative ionization mode were confirmed using tandem mass spectrometry wherein the fragmentation pattern and retention times of the parent ions in the tissue extract were matched with the standard compound. CID – collision-induced dissociation.

**Table 3:** Mass spectrometry confirmed identify of selected metabolites from ^56^Fe irradiation.

Metabolites (Kegg ID)	Mode	m/z	RT	p-value	Major CID fragments
Leucyl-leucine (C11332)	POS	245.186	2.3	↓ (0.02)	86.0949
Pyruvic acid (C00022)	POS	89.024	0.4	↑ (0.05)	70.0302, 66.4544
Prostaglandin E2 (C00584)	NEG	351.217	4.36	↑ (0.05)	333.2076, 271.2061
Uridine (C00299)	POS	245.078	0.4	↑ (0.04)	133.0439, 113.0356
Dethiobiotin (C01909)	POS	215.139	0.5	↓ (0.02)	197.1279, 109.1019,
					179.1171
Oxidized glutathione (C00127)	POS	613.162	0.4	↓ (0.02)	484.1080, 355.0681,
					231.0416
Glycyl-leucine (C02155)	NEG	187.109	0.5	↓ (0.01)	130.0872, 73.0407
*S-ATPA ( C13733)	NEG	227.097	1.2	↑ (0.03)	72.0053, 183.1127

Identity of biologically relevant selected metabolites from positive and negative mode were confirmed using tandem mass spectrometry wherein the fragmentation pattern and retention times of the parent ions in the tissue extract were matched with the standard compound. *(S)-α-Amino-3-hydroxy-5-t-butyl-4-isoxazolepropionic acid. CID – collision-induced dissociation

### Greater number of canonical pathways was perturbed with increased number of cancer related biomarkers upregulated after ^56^Fe radiation

**Figure 5:**
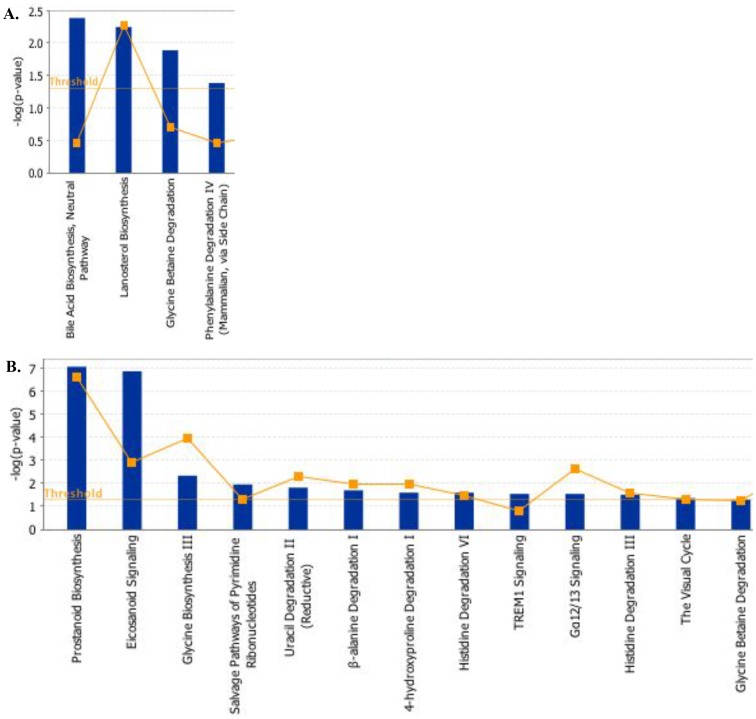
Metabolites from γ and ^56^Fe-irradiated groups were associated with distinctly different canonical pathways identified by Ingenuity Pathway Analysis. A) Metabolites from γ-irradiated groups were associated with four significantly perturbed canonical pathways (p<0.05 indicated by threshold line). B) Metabolites from ^56^Fe-irradiated groups were associated with thirteen significantly perturbed canonical pathways (p<0.05). Yellow line denotes –log(p-value) threshold of 1.3 which corresponds to p-value of 0.05.

Metabolites from the γ-irradiated group mapped to four IPA canonical pathways, which were significantly (p<0.05 which corresponds to -log(p-value)>1.3) perturbed (**Figure 5A** **and** **Table 4**). However, significant (p<0.05 which corresponds to -log(p-value)>1.3) perturbation of thirteen canonical pathways was identified by the IPA from the ^56^Fe irradiated dataset (**Figure 5B** **and top four presented in** **Table 4**). While the top pathway perturbed by the γ radiation was related to bile acid biosynthesis (**Figure 5A**), the top pathway in the ^56^Fe-irradiated group was prostanoid biosynthesis, which involves synthesis of prostaglandins (**Figure 5B**). Top biological functions delineated by the IPA from the γ-irradiated dataset were related to GI disease, hepatic system disease, infectious disease, and amino acid metabolism (**Table 5**). However, exposure to ^56^Fe radiation led to identification of biological functions related to inflammatory response, and cell growth and proliferation (**Table 5**). Exposure to γ and ^56^Fe radiation resulted in upregulation of six known biomarkers of human diseases in each radiation type (**Table 6**). In the γ-irradiated dataset, dysregulation of a number of metabolites including 17-hydroxyprogesterone, creatinine, dihydrotestosterone, glutathione, sarcosine, and ursodeoxycholic acid was found, which have been reported as biomarkers of a number of human diseases including prostate cancer [42]–[50] (**Table 6**). In the ^56^Fe-irradiated dataset, a number biomarkers such as 15-keto-13,14-dihydroprostaglandin E2 and PGE2 which are known to be associated with intestinal inflammatory disease and colon cancer [51]–[53] were identified by the IPA (**Table 6**). Additionally, IPA identified biomarkers epoprostenol, pyruvic acid, and thromboxane A2 have previously been reported to be associated with human cancers [54]–[57] (**Table 6**).

**Table 4:** Top four canonical pathways mapped by Ingenuity Pathway Analysis (IPA).

Canonical pathways	-log(p-value)	Molecules
**γ-ray**		
Bile acid biosynthesis,	2.38	7alpha-hydroxycholesterol,
Neutral Pathway		3alpha, 7alpha-dihydroxy-5beta-cholestane,
		7alpha-hydroxy-5beta-cholestan-3-one,
		3,7,12-trihydroxycoprostane,
		3,7,12-trihydroxycholestan-26-al,
		3alpha,7alpha-dihydroxy-5beta-cholestan-26-al,
		3alpha,7alpha-dihydroxy-5beta-cholestanic acid,
		7alpha,12alpha-dihydroxy-5beta-cholestan-3-one,
		3α,7α,12α, 26-tetrahydroxy-5beta-cholestane
Lanosterol biosynthesis	2.24	(S)-2,3-epoxysqualene, lanosterol
Glycine betaine	1.89	sarcosine, betaine, L-serine, L-methionine
degradation		
Phenylalanine	1.38	L-phenylalanine, phenylacetaldehyde,
degradation IV		phenylpyruvic acid, 3-phenyllactic acid,
(mammalian, via side		phenylacetic acid
chain)		
**^56^Fe**		
Prostanoid biosynthesis	7.08	prostaglandin H2, prostaglandin E2,
		epoprostenol, prostaglandin D2,
		thromboxane A2
Eicosanoid signaling	6.88	prostaglandin h2, prostaglandin E2, A4,
		epoprostenol, prostaglandin D2, lipoxin
		thromboxane A2, lipoxin B4
Glycine biosynthesis III	2.34	pyruvic acid, glyoxylic acid
Salvage pathways of	1.95	uridine, uracil, cytosine
pyrimidine		
ribonucleotides		

Significantly altered canonical pathways were obtained from metabolomics datasets using IPA and top four pathways are presented here along with corresponding p-value (-log) and molecules involved.

**Table 5:** Top five biological functions identified using Ingenuity Pathway Analysis.

A. Diseases and disorders		
Radiation	Name	# Molecules
γ-ray	Skeletal and muscular disorders	9
	Neurological disease	11
	Gastrointestinal disease	11
	Hepatic system disease	7
	Infectious disease	7
^56^Fe	Inflammatory response	19
	Hematological disease	7
	Immunological disease	6
	Inflammatory disease	4
	Respiratory disease	5
**B. Molecular and cellular functions**		
γ-ray	Amino acid metabolism	17
	Molecular transport	57
	Small molecule biochemistry	54
	Cell death and survival	52
	Gene expression	39
^56^Fe	Cellular movement	12
	Cell morphology	14
	Cellular development	23
	Cellular growth and proliferation	31
	Drug metabolism	3

Significantly altered biological functions associated with the experimental data set were identified using IPA and are listed above with the number of molecules from the data set of each radiation type significantly (p<0.05) involved in a specific biological function.

**Table 6:** Human disease related biomarkers identified in the data set.

Molecule (Kegg ID)	Fold Change			Biomarker applications
γ-ray				
17-hydroxyprogesterone		1.72	*Diagnosis*: breast cancer, polycystic ovarian	
(C01176)			syndrome	
Creatinine			*Treatment efficacy*: adrenal hyperplasia	
(C00791)		1.53	*Diagnosis*: acute renal failure, glucose	
			intolerance	
			*Treatment efficacy*: colorectal cancer,	
			Parkinson's disease, androgenic alopecia, Fabry	
			disease, osteoporosis	
			*Prognosis*: chronic kidney disease	
Dihydrotestosterone		1.58	*Diagnosis*: polycystic ovarian syndrome	
(C03917)			*Treatment efficacy*: prostate cancer, breast	
			cancer, androgenic alopecia	
Glutathione		308.09	*Diagnosis*: glaucoma, bladder cancer	
(C00051)			*Treatment efficacy*: atherosclerosis, alcoholic	
			liver disease	
Sarcosine		1.58	*Diagnosis*: prostate cancer	
(C00213)				
Ursodeoxycholic acid		1.51	*Diagnosis*: colorectal cancer	
(C07880)				
^56^Fe				
15-keto-13,14-		1.53	*Diagnosis*: ulcerative colitis	
dihydroprostaglandin E2			*Treatment efficacy*: lung cancer	
(C04671)				
Epoprostenol (C01312)		1.53	*Treatment efficacy*: breast cancer	
Prostaglandin E2		1.53	*Diagnosis*: Crohn's disease	
(C00584)			*Treatment efficacy*: lung cancer, head and neck	
			cancer, cervical cancer, esophageal cancer,	
			colorectal cancer, breast cancer	
Pyruvic acid		1.68	*Treatment efficacy*: brain cancer	
(C00022)				
Sarcosine		1.55	*Diagnosis*: prostate cancer	
(C00213)				
Thromboxane A2		1.53	*Treatment efficacy*: breast cancer	
(C02198)				

Different types of radiation exposure were associated with perturbation of metabolites known to be markers of a number of human diseases. Fold change for each metabolite in each radiation type is presented relative to sham-irradiated controls.

### Metabolite alterations were associated with proliferative pathway activation and intestinal epithelial cell proliferation after radiation exposure

**Figure 6:**
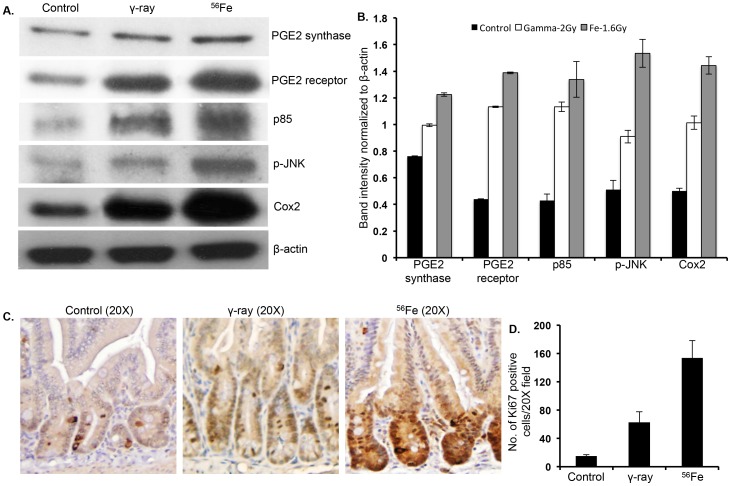
Greater activation of PGE2 dependent signaling pathways and increased proliferation in intestinal epithelial cell after ^56^Fe radiation. A) Immunoblots showing increased levels of PGE2 synthase, PGE2 receptor, PI3K (p85), phospho-JNK, and Cox2 two months after ^56^Fe radiation exposure. B) Quantification of immunoblots by normalizing band intensity to β-actin showed greater increase of specific proteins in ^56^Fe irradiated samples. C) Immunostaining for Ki67 showed increased number of positively stained cells in 56Fe irradiated groups relative to control and γ radiation groups. D) Quantification of Ki67 immunostaining showed significantly higher staining in ^56^Fe irradiated samples relative to γ irradiation.

Our study revealed that exposure to ^56^Fe radiation caused upregulation of ‘prostanoid biosynthesis’ and ‘ecosanoid signaling’, and increased PGE2 levels in the intestinal tissues. Immunoblot analysis of intestinal tissue lysate showed significantly increased levels of PGE2 synthase as well as upregulation of PGE2 receptor after ^56^Fe radiation relative to control and γ radiation (**Figure 6A and B**). Immunoblot analysis also showed higher levels of PI3K (p85), phospho-JNK, and Cox2, which are all downstream to PGE2 signaling (**Figure 6A and B**). We also observed, although less than ^56^Fe radiation, increased levels of PGE2 synthase, PGE2 receptor, PI3K (p85), phospho-JNK, and Cox2 in γ irradiated samples relative to controls (**Figure 6A and B** **)**. Immunostaining of intestinal section for Ki67, a marker of cell proliferation, showed significantly higher Ki67 positive cells in ^56^Fe irradiated samples relative to control and γ irradiated samples (p<0.0004 compared to control, p<0.02 compared to γ radiation; **Figure 6C and D**). Statistically significant increase in Ki67 positive cells was also observed after γ radiation relative to controls (p<0.003; **Figure 6C and D** **)**.

## Discussion

Exposure to ionizing radiation (IR) at non-lethal doses initiates a cascade of complex cellular response, which others and we have reported to be a continuous process [11],[58]–[61]. Additionally, the chronic cellular response after radiation exposure has been reported to be dependent on radiation quality [11],[58]–[60]. Heavy ion radiation in radiobiological terms is high-LET and hence is more damaging than low-LET γ-ray and x-ray radiation and is predicted to be a major risk factor to astronauts’ health during prolonged space missions [62]–[64]. Complete spectrum of long-term metabolic alterations after heavy ion radiation exposure in rapidly proliferating tissues such as intestine has not been examined in detail earlier. Indeed, this is the first report on persistent changes in intestinal tissue metabolites after heavy ion as well as γ radiation exposures. Here we demonstrate that exposure to low-LET γ and high-LET ^56^Fe radiation led to distinct intestinal tissue metabolic profiles. While exposure to ^56^Fe radiation was associated with increased levels of products from metabolic pathways related to inflammation, exposure to γ radiation led to increased metabolites related to bile acid biosynthesis. We further demonstrated, using immunoblots and immunohistochemistry, that ^56^Fe radiation-induced enhanced inflammatory metabolic pathways and consequent increased levels of inflammatory metabolites led to activation of proliferative signaling pathways in mouse intestine.

Metabolomics has emerged as an important tool in understanding radiation-induced adverse consequences in normal tissues. Acute metabolic response after exposure to different γ radiation doses has been reported in serum, urine, and in intestinal tissues for biomarker identification and biodosimetry [16],[20],[22],[23],[65],[66]. Given the importance of radiotherapy in cancer treatment and increasing interest in space travel and ongoing plan for mission to Mars, the urgency to understand the late effects of radiation on tissue metabolism is emerging as priority research area in radiation biology. We demonstrate that two months after exposure to γ as well as ^56^Fe radiation not only led to chronic perturbation of intestinal tissue metabolites but also led to radiation quality-dependent spatial separation of the metabolic profiles from each other and from the sham-irradiated control groups. This is suggestive of a persistent radiation response that is distinct for a given radiation type. Maintenance of physiologic and metabolic homeostasis in the GI tract is essential for nutritional wellbeing of the patient undergoing radiotherapy and therapeutic radiation exposure has been reported to cause long-term perturbation of metabolite transport across cell membranes as well as intracellular anabolic and catabolic pathways [67],[68]. Decrease in adenine, and guanosine was observed in mouse intestinal tissues two months after 2 Gy γ radiation, a dose commonly used is fractionated radiotherapy, is indicative of depletion in nucleotide pool. Depleted nucleotide pool, we envisage, could either be due to increased metabolic degradation or be due to decreased synthesis. However, increased levels of uridine and that of inosine support our belief of increased metabolic degradation of nucleotides. Nonetheless, reduced nucleotide pool not only heralds depleted energy store but it also affects repair processes of damaged DNA leading on one hand to diminutive effects on immune surveillance system and on the other to increasing probability of mutagenesis [69],[70]. Radiation exposure is known to cause DNA damage and heavy ion radiation has been shown to induce greater damage to DNA compared to γ radiation [11],[12]. When considered along with the fact that an effective nucleotide pool is essential for DNA replication and repair especially in rapidly proliferating tissues such as intestine [71], our results are suggestive of stress and perturbed homeostasis in the intestinal epithelial cells after radiation exposure with enhanced response after ^56^Fe radiation.

Radiation exposure has been reported to alter amino acid metabolism; however most of these studies were short term and none have studied heavy ion radiation [72],[73]. D-amino acids have been proposed to act as neurotransmitters in brain as well as in peripheral nerves and D-aspartate is reported to act via N-methyl-D-aspartate (NMDA) receptors [74]–[76]. Importantly, NMDA receptors are present on entroendocrine cells as well as on peripheral nerves involved in intestinal motility [74],[77] and our results showing decreased D-aspartate after γ radiation could affect normal intestinal function through decreased peristalsis as well as digestive enzyme production. Furthermore, because it is an endogenously produced amino acid, D-aspartate when decreased could suggest lower activity of metabolic pathway such as tricarboxylic acid (TCA) cycle and thus depleted energy source. We found the levels of 5-hydroxytrytophan (5-HTP) as well as phenylalanine to be significantly increased after γ radiation lending credence to dysregulated amino acid metabolism. While 5-HTP is generated from tryptophan by tryptophan hydroxylase, it is converted to serotonin by decarboxylases. Buildup of 5-HTP in γ-irradiated samples could be attributed to decreased carboxylase activity leading to reduced serotonin levels. Importantly, altered serotonin levels have been implicated in GI pathologies [78]. Elevated phenylalanine level could result from dysregulated phenylalanine hydroxylase activity and could lead to a deficiency state for a number of neurotransmitters including dopamine and serotonin as well as for the catecholamines - norepinephrine and epinephrine [79]. It is important to note here that while γ radiation exposure affected amino acids related to neurohormonal activity, the ^56^Fe radiation exposure led to altered dipeptide absorption. Decreased glycyl-leucine and increased leucyl-leucine dipeptides in the intestinal tissues after heavy ion radiation led us to believe that either dipeptide transport across intestinal epithelial cell is perturbed or function of dipeptide specific dipeptidase is altered. Taken together, our results indicate marked perturbation of amino acid metabolism after radiation exposure resulting in altered intestinal movement and dysregulated nutrient absorption [78],[80]–[82]. Dysregulated amino acid absorption is also evident from the fact that there are increased methionine and acetyltyrosne levels. Increased N-acetylmethionine (NAM) as well as L-carnitine observed after radiation exposure, we believe, is linked to increased methionine, which is rapidly acetylated in the cell [83] and methionine along with amino acid lysine can be used to synthesize L-carnitine [84]. Deficiency in aminoacylase 1, which converts NAM to methionine, has been associated with human diseases [83] and increased L-carnitine, which can be converted to trimethylamine-N-oxide (TMAO), has been linked to atherosclerosis in mice [85]. Importantly, although our results showed amino acid metabolism changes in both the radiation types, exposure to ^56^Fe radiation led to distinct as well as pronounced changes relative to γ radiation.

Exposure to radiation not only led to metabolic profiles that were distinct in γ and ^56^Fe radiation and radiation type dependent spatial separation of scores, but it also resulted in the identification of additional important molecular markers for human health risks. Exposure to γ radiation led to increased abundance, compared to control, of ursodeoxycholic acid, nicotinic acid, 3-ureidopropionate, 3α,7α Dihydroxy-5beta-cholestan-26-al, and creatinine. Importantly, levels of these metabolites remained unchanged in ^56^Fe irradiated samples relative to controls. Two of the metabolites, ursodeoxycholic acid and 3α,7α Dihydroxy-5beta-cholestan-26-al, are bile acid related and concur with increased ‘bile acid biosynthesis’, the top IPA-identified canonical pathway. Increased ‘bile acid biosynthesis’ was further supported by increased level of nicotinic acid observed after γ radiation [86]. Increased 3-ureidopropionate, an intermediate metabolite of uracil degradation, is consistent with decreased nucleotide pool discussed earlier. In contrast to γ radiation results, ^56^Fe-irradiation resulted in identification of additional metabolites, PGE2, pyruvate, glutamine, N6-acetyl-L-lysine, and L-histidine, which have profound implications for intestinal health and hence space explorers’ nutritional wellbeing. While L-histidine has been reported to possess anti-inflammatory function [87] and glutamine is essential for maintenance of intestinal mucosal health [88], N6-acetyl-L-lysine is known to play important roles in chromatin remodeling and hence gene function [89]. We believe that decreased L-histidine and glutamine along with increased PGE2, a pro-inflammatory molecule, could work in tandem to promote intestinal inflammatory response after ^56^Fe radiation. Furthermore, while decreased N6-acetyl-L-lysine herald heterochromatin and decreased gene expression, increased pyruvate denotes an altered metabolic state of acidosis [90],[91] after ^56^Fe radiation. Canonical pathway analysis in IPA of ^56^Fe dataset showed upregulation of ‘eicosanoid signaling’ and ‘prostanoid biosynthesis’ and both are involved in inflammatory response in intestinal mucosa [92],[93]. Eicosanoids are derived from fatty acids and consists of prostaglandins, prostacyclins, thromboxanes, lipoxins and leukotrienes and most of the members of these families of compounds produced by either cyclooxygenases or lipooxygenases are involved in pro-inflammatory responses. Indeed PGE2, and Thromboxane A2 are the two major biomarkers identified by the IPA in the ^56^Fe irradiated samples and both have been implicated in inflammatory pathologies in intestine including ulcerative colitis [92],[93]. Increased plasma levels of 15-keto-13, 14-dihydroprostaglandin E2, a major PGE2 metabolite detected in the ^56^Fe irradiated samples, have been used as a serum marker of PGE2 activity in tissues. Furthermore, elevated PGE2 due to its pro-inflammatory role and activation of downstream proliferative pathways such as PI3K/Akt, β-catenin, and JNK has been implicated in colorectal carcinogenesis and blocking PGE2 production has been reported to provide protection against colon cancer [51],[94]. Indeed, upon further probing of the metabolomic results using immunoblots, we demonstrated activation of proliferative pathways downstream of PGE2 after heavy ion radiation. Although metabolomic analysis did not detect PGE2 after γ radiation, we did observe, albeit less than ^56^Fe radiation, increased levels of molecules participating in PGE2 downstream pathways indicating potential long-term risk after exposure to both the radiation types. Metabolomic data showing increased PGE2 was further supported by upregulation of PGE2 synthesizing enzyme PGE2 synthase and we believe this is the first report showing a validated pathway-based long-term comparative study of intestinal tissue response after heavy ion and γ radiation exposure. When considered with our previously reported increased intestinal tumorigenesis in APC^Min/+^ mice after radiation exposure [95], our current results lead us to believe that exposures to a clinically relevant γ radiation dose and 1.6 Gy ^56^Fe radiation are potential risk factors for intestinal pathologies including CRC with greater risk after ^56^Fe radiation. Knowledge of molecular events occurring long after exposure to qualitatively different types of radiation is essential for devising strategies to minimize human health consequences associated with radiotherapy and space travel. The current study, while suggestive of a link between radiation exposure and intestinal pathologies, have also identified in each radiation type a number of key differential metabolites that can be used as a starting point to further evaluate them as long-term terrestrial as well as space radiation-risk biomarkers.

## Supporting Information

Figure S1 **Scores plot showing distinct metabolic changes and class separation in γ irradiated relative to sham irradiated mice in positive ionization mode.**
(JPG)

Figure S2 **Scores plot showing distinct metabolic changes and class separation in ^56^Fe irradiated mice relative to sham irradiation in positive ionization mode.**
(JPG)

## References

[1] Hamilton SA, Pecaut MJ, Gridley DS, Travis ND, Bandstra ER, et al (2006) A murine model for bone loss from therapeutic and space-relevant sources of radiation. J Appl Physiol 101: 789–793. 16741258 10.1152/japplphysiol.01078.2005

[2] Hayatsu K, Hareyama M, Kobayashi S, Yamashita N, Sakurai K, et al (2009) HZE Particle and Neutron Dosages from Cosmic Rays on the Lunar Surface. J. Phys. Soc. Jpn. 78: 149–152.

[3] Brooks A, Bao S, Rithidech K, Couch LA, Braby LA (2001) Relative effectiveness of HZE iron-56 particles for the induction of cytogenetic damage in vivo. Radiat Res 155: 353–359. 11175671 10.1667/0033-7587(2001)155[0353:reohip]2.0.co;2

[4] Curtis SB, Townsend LW, Wilson JW, Powers-Risius P, Alpen EL, et al (1992) Fluence-related risk coefficients using the Harderian gland data as an example. Adv Space Res 12: 407–416. 11537038 10.1016/0273-1177(92)90137-m

[5] Datta K, Neumann RD, Winters TA (2005) Characterization of complex apurinic/apyrimidinic-site clustering associated with an authentic site-specific radiation-induced DNA double-strand break. Proc Natl Acad Sci U S A 102: 10569–10574. 16024726 10.1073/pnas.0503975102 PMC1180784

[6] Hada M, Sutherland BM (2006) Spectrum of complex DNA damages depends on the incident radiation. Radiat Res 165: 223–230. 16435920 10.1667/rr3498.1

[7] Cucinotta FA, Wu H, Shavers MR, George K (2003) Radiation dosimetry and biophysical models of space radiation effects. Gravit Space Biol Bull 16: 11–18. 12959127

[8] Farthmann EH, Imdahl A, Eggstein S (1994) Radiation enteropathy. Strahlenther Onkol 170: 437–440. 8085209

[9] Johnson LB, Riaz AA, Adawi D, Wittgren L, Back S, et al (2004) Radiation enteropathy and leucocyte-endothelial cell reactions in a refined small bowel model. BMC Surg 4: 10. 15363103 10.1186/1471-2482-4-10 PMC522820

[10] Rosen IB, Shapiro BJ (1964) Radiation enteropathy of the small bowel. Can Med Assoc J 91: 681–688. 14201261 PMC1927638

[11] Datta K, Suman S, Kallakury BV, Fornace AJJ (2012) Exposure to heavy ion radiation induces persistent oxidative stress in mouse intestine. PLoS One 7: e42224. 22936983 10.1371/journal.pone.0042224 PMC3427298

[12] Datta K, Suman S, Trani D, Doiron K, Rotolo JA, et al (2012) Accelerated hematopoietic toxicity by high energy (56)Fe radiation. Int J Radiat Biol 88: 213–222. 22077279 10.3109/09553002.2012.639434 PMC3580183

[13] Suman S, Datta K, Trani D, Laiakis EC, Strawn SJ, et al. (2012) Relative biological effectiveness of (12)C and (28)Si radiation in C57BL/6J mice. Radiat Environ Biophys 10.1007/s00411-012-0418-9 PMC4208103 22562428

[14] Patterson AD, Li H, Eichler GS, Krausz KW, Weinstein JN, et al (2008) UPLC-ESI-TOFMS-based metabolomics and gene expression dynamics inspector self-organizing metabolomic maps as tools for understanding the cellular response to ionizing radiation. Anal Chem 80: 665–674. 18173289 10.1021/ac701807v PMC2254319

[15] Okunieff P, Chen Y, Maguire DJ, Huser AK (2008) Molecular markers of radiation-related normal tissue toxicity. Cancer Metastasis Rev 27: 363–374. 18506399 10.1007/s10555-008-9138-7 PMC2800946

[16] Coy SL, Cheema AK, Tyburski JB, Laiakis EC, Collins SP, et al (2011) Radiation metabolomics and its potential in biodosimetry. Int J Radiat Biol 87: 802–823. 21692691 10.3109/09553002.2011.556177 PMC3572797

[17] Ressom HW, Xiao JF, Tuli L, Varghese RS, Zhou B, et al (2012) Utilization of metabolomics to identify serum biomarkers for hepatocellular carcinoma in patients with liver cirrhosis. Anal Chim Acta 743: 90–100. 22882828 10.1016/j.aca.2012.07.013 PMC3419576

[18] Sheikh KD, Khanna S, Byers SW, Fornace AJ, Cheema AK (2011) Small molecule metabolite extraction strategy for improving LC/MS detection of cancer cell metabolome. J Biomol Tech 22: 1–4. 21455475 PMC3059537

[19] Kaur P, Rizk N, Ibrahim S, Luo Y, Younes N, et al (2013) Quantitative metabolomic and lipidomic profiling reveals aberrant amino acid metabolism in type 2 diabetes. Mol Biosyst 9: 307–317. 23247761 10.1039/c2mb25384d

[20] Ghosh SP, Singh R, Chakraborty K, Kulkarni S, Uppal A, et al (2013) Metabolomic changes in gastrointestinal tissues after whole body radiation in a murine model. Mol Biosyst 9: 723–731. 23403731 10.1039/c3mb25454b PMC3601576

[21] Patterson AD, Lanz C, Gonzalez FJ, Idle JR (2010) The role of mass spectrometry-based metabolomics in medical countermeasures against radiation. Mass Spectrom Rev 29: 503–521. 19890938 10.1002/mas.20272 PMC3690279

[22] Tyburski JB, Patterson AD, Krausz KW, Slavik J, Fornace AJJ, et al (2008) Radiation metabolomics. 1. Identification of minimally invasive urine biomarkers for gamma-radiation exposure in mice. Radiat Res 170: 1–14. 18582157 10.1667/RR1265.1 PMC2443732

[23] Tyburski JB, Patterson AD, Krausz KW, Slavik J, Fornace AJJ, et al (2009) Radiation metabolomics. 2. Dose- and time-dependent urinary excretion of deaminated purines and pyrimidines after sublethal gamma-radiation exposure in mice. Radiat Res 172: 42–57. 19580506 10.1667/RR1703.1 PMC2794378

[24] Klaunig JE, Xu Y, Isenberg JS, Bachowski S, Kolaja KL, et al (1998) The role of oxidative stress in chemical carcinogenesis. Environ Health Perspect 106 Suppl 1: 289–295. 9539021 10.1289/ehp.98106s1289 PMC1533298

[25] Garrett WS, Gordon JI, Glimcher LH (2010) Homeostasis and inflammation in the intestine. Cell 140: 859–870. 20303876 10.1016/j.cell.2010.01.023 PMC2845719

[26] Podolsky DK (1991) Inflammatory bowel disease (1). N Engl J Med 325: 928–937. 1881418 10.1056/NEJM199109263251306

[27] Podolsky DK (1991) Inflammatory bowel disease (2). N Engl J Med 325: 1008–1016. 1886623 10.1056/NEJM199110033251406

[28] Dey I, Lejeune M, Chadee K (2006) Prostaglandin E2 receptor distribution and function in the gastrointestinal tract. Br J Pharmacol 149: 611–623. 17016496 10.1038/sj.bjp.0706923 PMC2014644

[29] Sheng H, Shao J, Morrow JD, Beauchamp RD, DuBois RN (1998) Modulation of apoptosis and Bcl-2 expression by prostaglandin E2 in human colon cancer cells. Cancer Res 58: 362–366. 9443418

[30] Liu T, Laidlaw TM, Feng C, Xing W, Shen S, et al (2012) Prostaglandin E2 deficiency uncovers a dominant role for thromboxane A2 in house dust mite-induced allergic pulmonary inflammation. Proc Natl Acad Sci U S A 109: 12692–12697. 22802632 10.1073/pnas.1207816109 PMC3411985

[31] Sandig H, Pease JE, Sabroe I (2007) Contrary prostaglandins: the opposing roles of PGD2 and its metabolites in leukocyte function. J Leukoc Biol 81: 372–382. 17043246 10.1189/jlb.0706424

[32] Tessner TG, Muhale F, Schloemann S, Cohn SM, Morrison AR, et al (2004) Ionizing radiation up-regulates cyclooxygenase-2 in I407 cells through p38 mitogen-activated protein kinase. Carcinogenesis 25: 37–45. 14514662 10.1093/carcin/bgg183

[33] Obenaus A, Huang L, Smith A, Favre CJ, Nelson G, et al (2008) Magnetic resonance imaging and spectroscopy of the rat hippocampus 1 month after exposure to 56Fe-particle radiation. Radiat Res 169: 149–161. 18220468 10.1667/RR1135.1

[34] Peng Y, Brown N, Finnon R, Warner CL, Liu X, et al (2009) Radiation leukemogenesis in mice: loss of PU.1 on chromosome 2 in CBA and C57BL/6 mice after irradiation with 1 GeV/nucleon 56Fe ions, X rays or gamma rays. Part I. Experimental observations. Radiat Res 171: 474–483. 19397448 10.1667/RR1547.1

[35] Tucker JD, Marples B, Ramsey MJ, Lutze-Mann LH (2004) Persistence of chromosome aberrations in mice acutely exposed to 56Fe+26 ions. Radiat Res 161: 648–655. 15161355 10.1667/rr3177

[36] Bradford MM (1976) A rapid and sensitive method for the quantitation of microgram quantities of protein utilizing the principle of protein-dye binding. Anal Biochem 72: 248–254. 942051 10.1016/0003-2697(76)90527-3

[37] Johnson CH, Patterson AD, Krausz KW, Lanz C, Kang DW, et al (2011) Radiation metabolomics. 4. UPLC-ESI-QTOFMS-Based metabolomics for urinary biomarker discovery in gamma-irradiated rats. Radiat Res 175: 473–484. 21309707 10.1667/RR2437.1 PMC3089756

[38] Sieber M, Hoffmann D, Adler M, Vaidya VS, Clement M, et al (2009) Comparative analysis of novel noninvasive renal biomarkers and metabonomic changes in a rat model of gentamicin nephrotoxicity. Toxicol Sci 109: 336–349. 19349640 10.1093/toxsci/kfp070 PMC4830225

[39] Zhou B, Wang J, Ressom HW (2012) MetaboSearch: tool for mass-based metabolite identification using multiple databases. PLoS One 7: e40096. 22768229 10.1371/journal.pone.0040096 PMC3387018

[40] Shillingford JM, Piontek KB, Germino GG, Weimbs T (2010) Rapamycin ameliorates PKD resulting from conditional inactivation of Pkd1. J Am Soc Nephrol 21: 489–497. 20075061 10.1681/ASN.2009040421 PMC2831854

[41] Skaland I, Janssen EA, Gudlaugsson E, Klos J, Kjellevold KH, et al (2007) Phosphohistone H3 expression has much stronger prognostic value than classical prognosticators in invasive lymph node-negative breast cancer patients less than 55 years of age. Mod Pathol 20: 1307–1315. 17917671 10.1038/modpathol.3800972

[42] Hunter MH, Sterrett JJ (2000) Polycystic ovary syndrome: it’s not just infertility. Am Fam Physician 62: 1079–88, 1090 10997532

[43] Drafta DS, Stroe E, Schindler EE, Teodosiu T, Gozariu L, et al (1981) Adrenal function in early and metastatic breast cancer: dexamethasone suppression of plasma cortisol. Endocrinologie 19: 115–121. 6789444

[44] Clemons M, Goss P (2001) Estrogen and the risk of breast cancer. N Engl J Med 344: 276–285. 11172156 10.1056/NEJM200101253440407

[45] Rule AD, Larson TS, Bergstralh EJ, Slezak JM, Jacobsen SJ, et al (2004) Using serum creatinine to estimate glomerular filtration rate: accuracy in good health and in chronic kidney disease. Ann Intern Med 141: 929–937. 15611490 10.7326/0003-4819-141-12-200412210-00009

[46] Goldberg RM, Sargent DJ, Morton RF, Fuchs CS, Ramanathan RK, et al (2004) A randomized controlled trial of fluorouracil plus leucovorin, irinotecan, and oxaliplatin combinations in patients with previously untreated metastatic colorectal cancer. J Clin Oncol 22: 23–30. 14665611 10.1200/JCO.2004.09.046

[47] Silfen ME, Denburg MR, Manibo AM, Lobo RA, Jaffe R, et al (2003) Early endocrine, metabolic, and sonographic characteristics of polycystic ovary syndrome (PCOS): comparison between nonobese and obese adolescents. J Clin Endocrinol Metab 88: 4682–4688. 14557441 10.1210/jc.2003-030617

[48] Pendyala L, Velagapudi S, Toth K, Zdanowicz J, Glaves D, et al (1997) Translational studies of glutathione in bladder cancer cell lines and human specimens. Clin Cancer Res 3: 793–798. 9815751

[49] Sreekumar A, Poisson LM, Rajendiran TM, Khan AP, Cao Q, et al (2009) Metabolomic profiles delineate potential role for sarcosine in prostate cancer progression. Nature 457: 910–914. 19212411 10.1038/nature07762 PMC2724746

[50] Hess LM, Krutzsch MF, Guillen J, Chow HH, Einspahr J, et al (2004) Results of a phase I multiple-dose clinical study of ursodeoxycholic Acid. Cancer Epidemiol Biomarkers Prev 13: 861–867. 15159320

[51] Castellone MD, Teramoto H, Williams BO, Druey KM, Gutkind JS (2005) Prostaglandin E2 promotes colon cancer cell growth through a Gs-axin-beta-catenin signaling axis. Science 310: 1504–1510. 16293724 10.1126/science.1116221

[52] Pugh S, Thomas GA (1994) Patients with adenomatous polyps and carcinomas have increased colonic mucosal prostaglandin E2. Gut 35: 675–678. 8200564 10.1136/gut.35.5.675 PMC1374755

[53] Maxwell WJ, Kelleher D, Keating JJ, Hogan FP, Bloomfield FJ, et al (1990) Enhanced secretion of prostaglandin E2 by tissue-fixed macrophages in colonic carcinoma. Digestion 47: 160–166. 1964656 10.1159/000200492

[54] Sugahara KN, Teesalu T, Karmali PP, Kotamraju VR, Agemy L, et al (2010) Coadministration of a tumor-penetrating peptide enhances the efficacy of cancer drugs. Science 328: 1031–1035. 20378772 10.1126/science.1183057 PMC2881692

[55] Lee MS, Moon EJ, Lee SW, Kim MS, Kim KW, et al (2001) Angiogenic activity of pyruvic acid in in vivo and in vitro angiogenesis models. Cancer Res 61: 3290–3293. 11309282

[56] Matsui Y, Amano H, Ito Y, Eshima K, Suzuki T, et al (2012) Thromboxane A(2) receptor signaling facilitates tumor colonization through P-selectin-mediated interaction of tumor cells with platelets and endothelial cells. Cancer Sci 103: 700–707. 22296266 10.1111/j.1349-7006.2012.02200.x PMC7659242

[57] Dassesse T, de Leval X, de Leval L, Pirotte B, Castronovo V, et al (2006) Activation of the thromboxane A2 pathway in human prostate cancer correlates with tumor Gleason score and pathologic stage. Eur Urol 50: 1021–31 discussion 1031 16522350 10.1016/j.eururo.2006.01.036

[58] Suman S, Johnson MD, Fornace AJJ, Datta K (2012) Exposure to ionizing radiation causes long-term increase in serum estradiol and activation of PI3K-Akt signaling pathway in mouse mammary gland. Int J Radiat Oncol Biol Phys 84: 500–507. 22381906 10.1016/j.ijrobp.2011.12.033 PMC3580184

[59] Rola R, Sarkissian V, Obenaus A, Nelson GA, Otsuka S, et al (2005) High-LET radiation induces inflammation and persistent changes in markers of hippocampal neurogenesis. Radiat Res 164: 556–560. 16187787 10.1667/rr3412.1

[60] Tseng B, Giedzinski E, Izadi A, Suarez T, Lan M, et al. (2013) Functional consequences of radiation-induced oxidative stress in cultured neural stem cells and the brain exposed to charged particle irradiation. Antioxid Redox Signal 10.1089/ars.2012.5134 PMC3936501 23802883

[61] Snyder AR, Morgan WF (2004) Gene expression profiling after irradiation: clues to understanding acute and persistent responses? Cancer Metastasis Rev 23: 259–268. 15197327 10.1023/B:CANC.0000031765.17886.fa

[62] Cucinotta FA, Schimmerling W, Wilson JW, Peterson LE, Badhwar GD, et al (2001) Space radiation cancer risks and uncertainties for Mars missions. Radiat Res 156: 682–688. 11604093 10.1667/0033-7587(2001)156[0682:srcrau]2.0.co;2

[63] Cucinotta FA, Durante M (2006) Cancer risk from exposure to galactic cosmic rays: implications for space exploration by human beings. Lancet Oncol 7: 431–435. 16648048 10.1016/S1470-2045(06)70695-7

[64] Durante M, Cucinotta FA (2008) Heavy ion carcinogenesis and human space exploration. Nat Rev Cancer 8: 465–472. 18451812 10.1038/nrc2391

[65] Laiakis EC, Hyduke DR, Fornace AJ (2012) Comparison of mouse urinary metabolic profiles after exposure to the inflammatory stressors gamma radiation and lipopolysaccharide. Radiat Res 177: 187–199. 22128784 10.1667/rr2771.1 PMC3286010

[66] Johnson CH, Patterson AD, Krausz KW, Kalinich JF, Tyburski JB, et al (2012) Radiation metabolomics. 5. Identification of urinary biomarkers of ionizing radiation exposure in nonhuman primates by mass spectrometry-based metabolomics. Radiat Res 178: 328–340. 22954391 10.1667/rr2950.1 PMC3498937

[67] Vistad I, Kristensen GB, Fossa SD, Dahl AA, Morkrid L (2009) Intestinal malabsorption in long-term survivors of cervical cancer treated with radiotherapy. Int J Radiat Oncol Biol Phys 73: 1141–1147. 18760883 10.1016/j.ijrobp.2008.05.064

[68] Caster WO, Armstrong WD (1956) Electrolyte metabolism after total-body x-irradiation. Radiat Res 5: 189–204. 13350499

[69] Snyder RD (1985) Effects of nucleotide pool imbalances on the excision repair of ultraviolet-induced damage in the DNA of human diploid fibroblasts. Basic Life Sci 31: 163–173. 3888172 10.1007/978-1-4613-2449-2_10

[70] Hess JR, Greenberg NA (2012) The role of nucleotides in the immune and gastrointestinal systems: potential clinical applications. Nutr Clin Pract 27: 281–294. 22392907 10.1177/0884533611434933

[71] Mathews CK, Sinha NK (1982) Are DNA precursors concentrated at replication sites? Proc Natl Acad Sci U S A 79: 302–306. 7043458 10.1073/pnas.79.2.302 PMC345714

[72] Liu H, Wang Z, Zhang X, Qiao Y, Wu S, et al (2013) Selection of candidate radiation biomarkers in the serum of rats exposed to gamma-rays by GC/TOFMS-based metabolomics. Radiat Prot Dosimetry 154: 9–17. 22951997 10.1093/rpd/ncs138

[73] Lee do Y, Bowen BP, Nguyen DH, Parsa S, Huang Y, et al (2012) Low-dose ionizing radiation-induced blood plasma metabolic response in a diverse genetic mouse population. Radiat Res 178: 551–555. 23051006 10.1667/RR2990.1

[74] Burns GA, Stephens KE (1995) Expression of mRNA for the N-methyl-D-aspartate (NMDAR1) receptor and vasoactive intestinal polypeptide (VIP) co-exist in enteric neurons of the rat. J Auton Nerv Syst 55: 207–210. 8801272 10.1016/0165-1838(95)00043-w

[75] Horio M, Ishima T, Fujita Y, Inoue R, Mori H, et al (2013) Decreased levels of free D-aspartic acid in the forebrain of serine racemase (Srr) knock-out mice. Neurochem Int 62: 843–847. 23439386 10.1016/j.neuint.2013.02.015

[76] Kim PM, Duan X, Huang AS, Liu CY, Ming GL, et al (2010) Aspartate racemase, generating neuronal D-aspartate, regulates adult neurogenesis. Proc Natl Acad Sci U S A 107: 3175–3179. 20133766 10.1073/pnas.0914706107 PMC2840285

[77] Fukunaga Y, Kimura M, Saitoh O (2010) NMDA receptor in intestinal enteroendocrine cell, STC-1. Neuroreport 21: 772–776. 20531233 10.1097/WNR.0b013e32833bfd17

[78] Costedio MM, Hyman N, Mawe GM (2007) Serotonin and its role in colonic function and in gastrointestinal disorders. Dis Colon Rectum 50: 376–388. 17195902 10.1007/s10350-006-0763-3

[79] Hyland K (2007) Inherited disorders affecting dopamine and serotonin: critical neurotransmitters derived from aromatic amino acids. J Nutr 137: 1568S–1572S discussion 1573S 17513427 10.1093/jn/137.6.1568S

[80] Hubel KA (1976) Intestinal ion transport: effect of norepinephrine, pilocarpine, and atropine. Am J Physiol 231: 252–257. 961867 10.1152/ajplegacy.1976.231.1.252

[81] Aulsebrook KA (1965) intestinal absorption of glucose and sodium: effects of epinephrine and norepinephrine. Biochem Biophys Res Commun 18: 165–169. 14282012 10.1016/0006-291x(65)90734-5

[82] Munro AF (1951) The effect of adrenaline on the guinea-pig intestine. J Physiol 112: 84–94. 14825182 10.1113/jphysiol.1951.sp004510 PMC1393056

[83] Smith T, Ghandour MS, Wood PL (2011) Detection of N-acetyl methionine in human and murine brain and neuronal and glial derived cell lines. J Neurochem 118: 187–194. 21554324 10.1111/j.1471-4159.2011.07305.x

[84] Steiber A, Kerner J, Hoppel CL (2004) Carnitine: a nutritional, biosynthetic, and functional perspective. Mol Aspects Med 25: 455–473. 15363636 10.1016/j.mam.2004.06.006

[85] Koeth RA, Wang Z, Levison BS, Buffa JA, Org E, et al (2013) Intestinal microbiota metabolism of L-carnitine, a nutrient in red meat, promotes atherosclerosis. Nat Med 19: 576–585. 23563705 10.1038/nm.3145 PMC3650111

[86] Holland RE, Rahman K, Morris AI, Coleman R, Billington D (1993) Effects of niacin on biliary lipid output in the rat. Biochem Pharmacol 45: 43–49. 8424822 10.1016/0006-2952(93)90375-7

[87] Peterson JW, Boldogh I, Popov VL, Saini SS, Chopra AK (1998) Anti-inflammatory and antisecretory potential of histidine in Salmonella-challenged mouse small intestine. Lab Invest 78: 523–534. 9605177

[88] van der Hulst RR, von Meyenfeldt MF, Deutz NE, Soeters PB (1997) Glutamine extraction by the gut is reduced in depleted [corrected] patients with gastrointestinal cancer. Ann Surg 225: 112–121. 8998127 10.1097/00000658-199701000-00013 PMC1190613

[89] Sterner DE, Berger SL (2000) Acetylation of histones and transcription-related factors. Microbiol Mol Biol Rev 64: 435–459. 10839822 10.1128/mmbr.64.2.435-459.2000 PMC98999

[90] Platt BS, Lu GD (1939) Studies on the metabolism of pyruvic acid in normal and vitamin B(1)-deficient states: The accumulation of pyruvic acid and other carbonyl compounds in beri-beri and the effect of vitamin B(1). Biochem J 33: 1525–1537. 16747062 10.1042/bj0331525 PMC1264610

[91] Gilliland IC, Martin MM (1951) Raised blood pyruvic acid level in diabetic acidosis; the value of cocarboxylase in treatment. Br Med J 1: 14–16. 14792078 10.1136/bmj.1.4696.14 PMC2068141

[92] Zifroni A, Treves AJ, Sachar DB, Rachmilewitz D (1983) Prostanoid synthesis by cultured intestinal epithelial and mononuclear cells in inflammatory bowel disease. Gut 24: 659–664. 6345283 10.1136/gut.24.7.659 PMC1420025

[93] Eberhart CE, Dubois RN (1995) Eicosanoids and the gastrointestinal tract. Gastroenterology 109: 285–301. 7797026 10.1016/0016-5085(95)90296-1

[94] Nakanishi M, Menoret A, Tanaka T, Miyamoto S, Montrose DC, et al (2011) Selective PGE(2) suppression inhibits colon carcinogenesis and modifies local mucosal immunity. Cancer Prev Res (Phila) 4: 1198–1208. 21576350 10.1158/1940-6207.CAPR-11-0188 PMC3151318

[95] Datta K, Suman S, Kallakury BV, Fornace AJJ (2013) Heavy ion radiation exposure triggered higher intestinal tumor frequency and greater beta-catenin activation than gamma radiation in APC(Min/+) mice. PLoS One 8: e59295. 23555653 10.1371/journal.pone.0059295 PMC3605451

