# Supplementary figures and images for: Long-Term Differential Changes in Mouse Intestinal Metabolomics after γ and Heavy Ion Radiation Exposure
Source: PLoS One. 2014 Jan 27;9(1):e87079. doi: 10.1371/journal.pone.0087079 (PMC3903607; doi:10.1371/journal.pone.0087079)

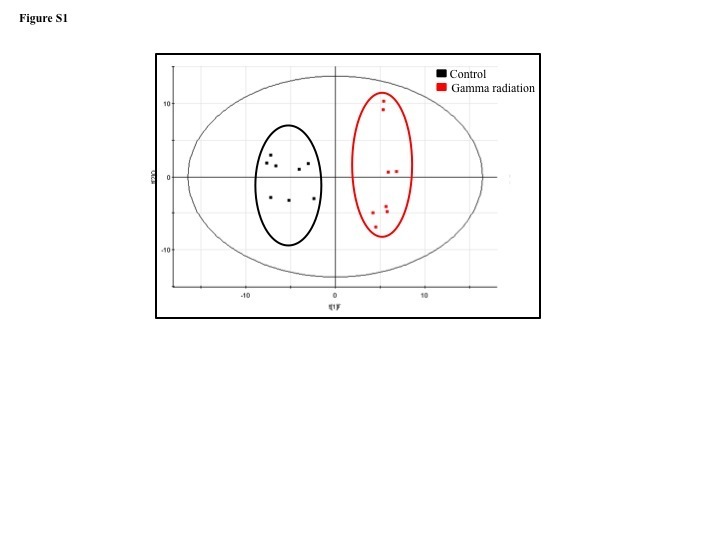

Supplement: Figure S1 — Scores plot showing distinct metabolic changes and class separation in γ irradiated relative to sham irradiated mice in positive ionization mode. (JPG) [file pone.0087079.s001.jpg]

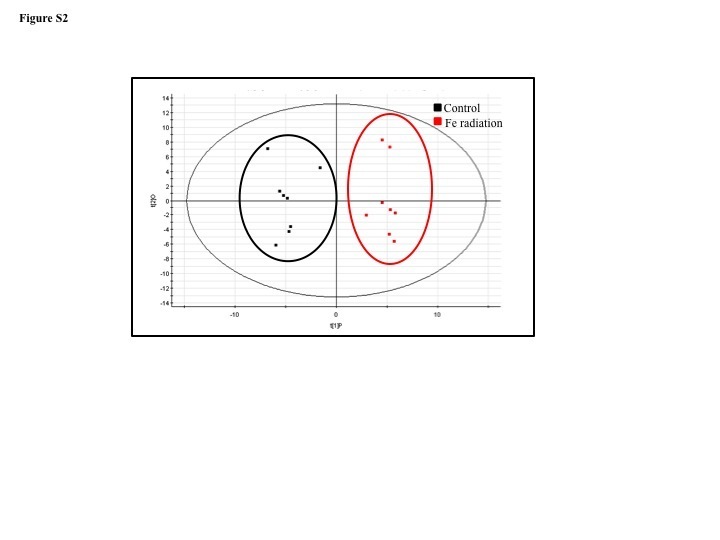

Supplement: Figure S2 — Scores plot showing distinct metabolic changes and class separation in 56Fe irradiated mice relative to sham irradiation in positive ionization mode. (JPG) [file pone.0087079.s002.jpg]
